# Chronic Non‐Progressive Neurophysiology in an ALS Patient Treated With Tofersen for 6 Years

**DOI:** 10.1002/mus.70417

**Published:** 2026-09-25

**Authors:** Ivar Winroth, Christian Krarup, Henrik Berntsson, Lisette Salvesen, Kirsten Svenstrup, Erik Nordh, Peter M. Andersen

**Affiliations:** ^1^ Department of Clinical Sciences, Neurosciences Umeå University Umeå Sweden; ^2^ Department of Clinical Neurophysiology Copenhagen University Hospital, Rigshospitalet, and University of Copenhagen Copenhagen Denmark; ^3^ Department of Neurology Halland Hospital Varberg Varberg Sweden; ^4^ Department of Neurology Copenhagen University Hospital, Bispebjerg Hospital Copenhagen Denmark; ^5^ Department of Neurology Copenhagen University Hospital, Rigshospitalet Copenhagen Denmark

**Keywords:** ALS, SOD1, tofersen

## 1 Introduction

Mutations in the gene encoding superoxide dismutase‐1 (SOD1) are a cause of amyotrophic lateral sclerosis (ALS), likely through a dose‐dependent toxic gain‐of‐function [1, 2]. Intrathecal treatment with tofersen, an antisense oligonucleotide (ASO) reducing *SOD1* expression, has been shown to lower neurofilament light chain (NfL) and stabilizing ALS Functional Rating Scale Revised (ALSFRS‐R) scores in many patients, but no study has included neurophysiological assessments [3, 4, 5, 6]. We here present an ALS case caused by the aggressive A4S *SOD1* mutation with a short expected survival that turned chronic and non‐progressive with tofersen treatment over 6 years [2, 7].

## 2 Case Report

The patient was a 34 year old male with an ALS family history over 3 generations and from presymptomatic DNA testing known heterozygous carrier of A4S *SOD1*. He was otherwise healthy. The first symptom (onset date; OD) was proximal weakness in his right leg, progressing to the right arm within weeks and muscle twitching in his arms and trunk. He was diagnosed with ALS at OD +3 months according to the European Federation of Neurological Societies (EFNS) guidelines [8] and started riluzole medication. He was enrolled in the phase‐3 VALOR study at OD +6 months, randomized 2:1 to tofersen 100 mg/4 weeks or placebo for 28 weeks, followed by 100 mg every 4 weeks [3].

He participated in other clinical investigations at OD +3 months prior to participation in the VALOR study and then again at OD, +51 months and + 68 months following completion of the trial. Clinical assessment, ALSFRS‐R, and NfL were collected regularly.

The patient received 81 injections of tofersen and was clinically stable. He has experienced no side effects except lumbar pain related to the procedure and asymptomatic pleocytosis with mononuclear cells. Due to pleocytosis at OD +1 year (monocyte range from 1 to 75 cells/μL), magnetic resonance imaging of the neuroaxis was performed showing no abnormal signal except for localized contrast enhancement at the site of the injections.

Clinical, laboratory and electrodiagnostic findings are summarized in Tables 1 and 2. At diagnosis, predominantly lower motor neuron (LMN) findings were observed in the right arm and leg only with mild signs of upper motor neuron (UMN) involvement in all four limbs. At OD +68 months, mild LMN findings were identified in the left lower limb. Between OD +3 and + 6 months, ALSFRS‐R decreased from 45 to 41, for example, a loss of 1.1 point per month assuming a linear decline and that the patient scored 48 before symptom onset. However, this progression has almost completely abated as ALSFRS‐R has been fluctuating around 38 ± 2 points since the end of the first year (Figure 1). Longitudinal cognitive assessment using ECAS has been unremarkable [9]. At diagnosis, CSF‐NfL was 11,000 pg/mL but has since decreased to the 780–1400 pg/mL range, supporting a greatly reduced disease activity.

**TABLE 1:** Summary of clinical features.

	OD +3 months (at diagnosis)	OD +50 months	OD +68 months	Normative values
ALSFRS‐R	45	35	39	48
ECAS	107	117	114	> 106
csf‐NfL	11,000	1710	1080	< 560 pg/mL
Plasma‐NfL	Not available	12	31	< 19 pg/mL
Plasma‐CK	324	648	666	< 402 IU/L
Plasma‐Troponin T	8	82	60	< 14.51 ng/L

Abbreviations: OD, onset date; ALSFRS‐R, ALS functional rating scale revised; ECAS, Edinburgh Cognitive And Behavioral ALS Screen; NfL, neurofilament light chain; CK, creatinine kinase.

**TABLE 2:** Neurophysiological findings.

	OD +3 months (Umeå, Sweden)	OD +50 months (Umeå, Sweden)	OD +52 months (Copenhagen, Denmark)
NCS upper extremities	Normal.	Normal, but CMAP amp relatively reduced. For example, right ulnar nerve CMAP amp reduced from 9.1 to 6.2 mV (4.0) since OD +3. Normal sensory NCS.	Distal latency right median nerve: 3.6 ms (3.4).
NCS lower extremities	CMAP amp left fibular nerve 1.2 mV (3.4). Other NCS normal.	CMAP amp left fibular nerve 0,7 mV (3.4). Other NCS normal, but CMAP amp relatively reduced, for example, right tibial nerve reduced from 15.8 to 7.7 mV (6.0) since OD +3.	Normal right fibular nerve.
EMG findings	Spontaneous activity (+) rDM, rEDC, rFDIH, rVL, bTA. MUP abnormalitites *Amplitude* (+) bTZ, bDM, rFDIH, rEDC, lTA + rVL, rTA *Duration* (+) bTZ, bDM, rFDIH, rEDC + rVL, rTA *Polyphasia* (+) bTZ, lDM, rEDC, rFDIH, rVL, bTA + rDM	Spontaneous activity (+) rDM, rBB, rFDIH, bVM, lTA + rTA ++ lMM MUP abnormalities *Amplitude* + lMM, rDM, rFDIH, rVM ++ rBB, lVM, bTA *Duration* + rDM, rBB, rFDIH, bVM, bTA ++ lMM *Polyphasia* (+) lMM, rVM, rTA + rDM, rBB, rFDIH, lTA *Normal EMG* lBB, lFDIH	Spontaneous activity + rAPB, rTA. MUP abnormalities *Amplitude* + rSCM ++ lDM, lVM, rTA *Duration* + rAPB ++ rSCM, lDM, lVM, rTA
Ultrasound	rTZ, rBB, rEDC, rVL, rTA, lVM with sonographic fasciculations.	rTZ, rDM, rBB, rAPB, rRA, rVL, rVM, rBF with sonographic fasciculations.	Not performed

*Note:* Parentheses denote normal limit where applicable. All nerves examined with NCS are not presented. All muscles examined with EMG are presented.

Abbreviations: (+), slight increase; +, moderate increase; ++, marked increase; APB, abductor pollicis brevis; BB, biceps brachii; b, bilateral; BF, biceps femoris; CMAP amp, compound muscle action potential amplitude; DM, Deltoid muscle; EDC, extensor digitorum; EMG, electromyography; FDIH, first dorsal interosseus of the hand; l, left; MEP, motor evoked potential by transcranial magnetic stimulation; MM, masseter muscle; MUP, motor unit potential; NCS, nerve conduction study; OD, onset date; RA, rectus abdominis; r, right; SCM, sternocleidomastoid muscle; TA, tibialis anterior; TZ, Trapezius; VL, vastus lateralis; VM, vastus medialis.

**FIGURE 1:**
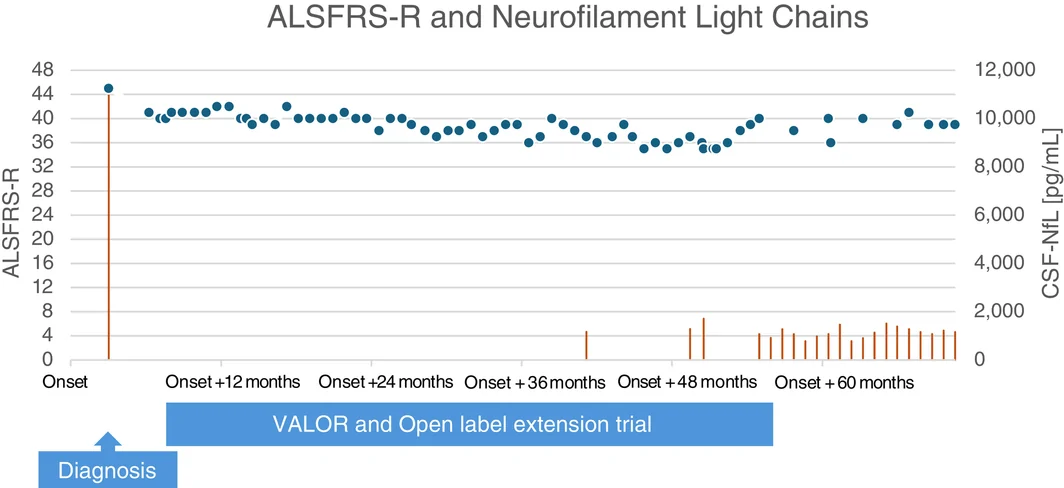
ALSFRS‐R score and CSF‐Neurofilament Light chain. ALSFRS‐R score is represented as blue dots and CSF Neurofilament Light chain level as orange vertical bars. The first recorded ALSFRS‐R and NfL were at the diagnostic work‐up 3 months after onset. The neurofilament and other biomarker data recorded during the VALOR and VALOR open label extension trial period are censored but are part of the data presented in references [3] and [6]. Since completion of the VALOR trial, the patient has received tofersen in an Early Access Program and as a prescription drug.

MScanFit motor unit number estimation (MUNE) demonstrated a low normal number of motor units (51–66) in the left abductor pollicis brevis (APB) muscle and a non‐progressive severely reduced number of motor units in the right APB, with a recent non‐meaningful increase from 14 at OD +60 months to 23 at OD +78 months.

## 3 Discussion

We here present longitudinal clinical neurophysiological data of a patient successfully treated with tofersen for 6 years. Tofersen has arrested the disease progression, demonstrated by a flattening of the ALSFRS‐R progression curve after the first year of treatment, near normalization of NfL and stabilization of the MUNE number. Although ALSFRS‐R progression in ALS is rarely linear, the observed change in disease progression constitutes a likely treatment effect. The six mutations in *SOD1* codon A4 are invariably associated with relentless progressive ALS disease leading to death with no reports of spontaneous remission in the literature since the first report of aggressive mutations in *SOD1* codon A4 in 1993. In VALOR, the serious adverse event rate was 7%, but this patient has tolerated tofersen well for years [3].

Although not all patients improve with tofersen, this patient had a number of potential beneficial prognostic factors. Firstly, treatment was started only 6 months after symptom onset. That early intervention may be associated with better tofersen response was demonstrated in the VALOR trial and in a recent Icelandic report on tofersen responders [3, 4]. Secondly, he was a young adult with no co‐morbidities. Thirdly, he carried a high‐risk mutation in *SOD1* codon A4 associated with an aggressive disease course and an estimated median survival from onset of paresis of 1–2 years [7, 10]. Hypothetically, an aggressive disease course would suggest that the underlying disease mechanism is still in its early stages at the time of diagnosis and may be more amenable to effective intervention compared to a slow disease course in which secondary effects of the disease may be more pronounced.

Longitudinal neurophysiological examinations demonstrated both subacute and chronic neurogenic changes in affected muscles. This, together with mildly elevated but fluctuating NfL level, indicates the presence of a low grade active disease component. However, no additional motor unit loss has been detected in the right hand since the first MUNE. Also, no clinical or neurophysiological sign of involvement of the distal left arm, truncal, or bulbar‐innervated myotomes has been observed. This supports the notion that tofersen may not only limit the progressive motor neuron loss in an already affected limb but also the spread to other myotomes. It is possible that the low grade disease activity in already affected extremities could constitute a focal dose‐dependent treatment failure.

The patient presented here represents a growing new population of people living and coping with hereditary ALS for longer periods of time, putatively denoted chronic ALS (in contrast to progressive ALS). The case demonstrates the effect of ASOs in the treatment of ALS and highlights the importance of early intervention. MUNE was generally correlative with the patient's clinical course and CSF‐NfL levels and could be a promising outcome measure in future treatment trials, but more research is needed.

## Author Contributions

**Ivar Winroth:** conceptualization, investigation, project administration, visualization, writing – original draft, writing – review and editing. **Christian Krarup:** investigation, writing – original draft, writing – review and editing. **Henrik Berntsson:** investigation, writing – review and editing. **Lisette Salvesen:** investigation, writing – review and editing. **Kirsten Svenstrup:** investigation, writing – review and editing. **Erik Nordh:** investigation, writing – review and editing. **Peter M. Andersen:** conceptualization, funding acquisition, investigation, project administration, writing – original draft, writing – review and editing.

## Funding

This work was supported by Hjärnfonden, 2020‐0353, 2022‐0309, 2025‐0362; Vetenskapsrådet, 2017‐03100; Knut och Alice Wallenbergs Stiftelse, 2012.0091, 2014.0305, 2020.0232, 2023.0460; ALS Foundation, 2025.18A; Neuroförbundet; Västerbotten County Council, RV1035297, RV1031455; Fort Knox Charity Foundation; Olsson and Olsson Charity Foundation.

## Ethics Statement

The authors confirm that we have read the Journal's position on issues involved in ethical publication and affirm that this report is consistent with those guidelines.

## Conflicts of Interest

Drs. Salvesen and Svenstrup were investigators in the VALOR trial. Dr. Andersen serves on the advisory board for the tofersen trials and his institution has received compensation for this service from Biogen. He is also a principal investigator in the ongoing ATLAS trial. He is also an investigator and serves on advisory boards for competing SOD1‐trials by uniQure and Regeneron and his institution has received financial compensation for this service. He was also the principal investigator in AL‐S Pharma's AP101‐02 trial testing an antibody against misfolded SOD1 protein. The other authors declare no conflicts of interest.

## Data Availability

The data that support the findings of this study are available on request from the corresponding author. The data are not publicly available due to privacy or ethical restrictions.
